# Community preventive behaviour and perception on the severity of COVID-19 disease in Indonesia, 2021-2022: Structural equation modelling

**DOI:** 10.12688/f1000research.135262.1

**Published:** 2023-08-10

**Authors:** Tris Eryando, Tiopan Sipahutar, Sandeep Poddar

**Affiliations:** 1 Department of Biostatistics and Population, Faculty of Public Health, Universitas Indonesia, Depok, West Java, 16424, Indonesia; 2 Research and Innovations, Lincoln University College,, Petaling Jaya, Selangor, 47301, Malaysia

**Keywords:** COVID-19; perceive; preventive behavior; Health Belief Model; Indonesia

## Abstract

**Background:** This study investigated the determinants of community preventive behavior in complying with the Indonesian regulations to prevent COVID-19 local transmission.

**Methods:** A cross-sectional study used to collect the data via an online cross using a form created from a google questionnaire forms. A total of 1,802 respondents were gathered at a single point in time. The authors used the Health Belief Model (HBM) approach to measure and create a model of preventive behavior for COVID-19.

**Results**: The findings showed that self-efficacy and perceived barriers had statistically significant relationships with preventive behavior. However, the goodness of fit index showed that the proposed model was not fit for the data, which means that it was not fit to describe the empirical phenomenon under study.

**Conclusions:** This study found that more than half of the respondents still had low perceived susceptibility and severity. Only a few respondents had significant barriers to implementing COVID-19 transmission prevention behaviors. Still, most respondents had low perceived self-efficacy, and only 60% had good behaviors related to COVID-19 prevention. We recommended increasing perceived susceptibility and severity by providing the correct information about COVID-19 in the local cultural context.

**Abbreviations**
A: Age
AVE: Average Variance Extracted
AGFI: Adjusted Goodness of Fit Index
BEN1-5: Items for perceive Benefit
BAR 1-5: Items for perceive Barrier
BHV1-6: Items for Behaviour
CFI: Comparative Fit Index
E: Education
G: Gender
GFI: Goods of Fit Index
HBM: Health Belief Model
IBM: International Business Machines
IFI: Incremental Fit Index
KAP: Knowledge Attitude Practice
MI: Modification Indices
NFI: Normed Fit Index
O: Occupation
PNFI: Parsimonious Normed Fit Index
PGFI: Parsimonious Goods of Fit Index
RFI: Relative Fir Index
R: Residence
RMSEA: Root Mean Square Error of Approximation
SEM: Structural Equation Model
SPSS: Statistical Package for The Social Sciences
SRMR: Standardized Root Mean Square
S1-5: Items for perceive Severity
SE1-5: Items for perceive Self-Efficacy
UNICEF: United Nations International Children Fund
WHO: World Health Organization
X1-14: Items for perceive Susceptibility
3M: Wearing mask, washing hand, and social distancing

## Introduction

The world is currently besieged by the COVID-19 pandemic. ^1^ As of February 7, 2022, there were 394,381,395 confirmed cases of COVID-19, with the World Health Organization (WHO) reporting 5,735,179 deaths. ^2^ The COVID-19 pandemic in Indonesia from mid-2021 until the end of 2021 reached its peak (second wave) when it was dominated by the Delta variant.

COVID-19 prevention regulations largely depend on community compliance and behavior. While changing behavior is a significant challenge in health interventions. An AC Nielsen survey (2020) in six major cities with a total of 2,000 respondents, in collaboration with the United Nations International Children’s Emergency Fund (UNICEF), found that less than one-third (31.5%) of all respondents practiced all three-preventive activities (wearing masks, washing hands, and social distancing). Over one-third (36%) practiced only two of the three-preventive behaviors, less than one-fourth (23.2%) practiced only one of the three behaviors, and almost one-tenth (9.3%) did nothing at all. ^3^ A number of studies described the associations between socio-demographic characteristics and people’s levels of perceptions about the severity of COVID-19 with their preventive behavior. ^3^^–^^10^

Based on the official government report (see https://covid19.go.id/monitoring-kepatuhan-protokol-kesehatan), some areas of Indonesia showed a compliance rate of only 60% till January 2022. ^11^ The aim of this study was to examine changes in community behaviour to prevent local COVID-19 transmissions and changes in community perceptions about the severity level of COVID-19. The following research questions were formulated: 1) What constitutes as the community perceptions of COVID-19? 2) Is there an association between community perception and community preventive behaviour using the Health Belief Model (HBM) approach? And 3) what kind of model of preventive behaviour on COVID-19 can be predicted using the structural equation model (SEM)?

## Methods

### Study design and samples

This was a cross-sectional study using Google forms with structured survey questionnaires. The questionnaires have been tested with 30 respondents before the survey was conducted. The proportion of a large population was used to figure out the sample size for a variable, which was used to figure out the minimum sample size. ^12^ The margin error=5%, p=50%, and Zα=95%. The minimum sample size for this study was 385 respondents. The data collection was conducted over two weeks from July 14–26, 2021, using WhatsApp, Line, and Telegram. To expand the coverage throughout Indonesia, social media influencers were asked to distribute the survey through Twitter and Instagram. The respondents who participated in this study were aged 15–62 and Indonesian citizens. On average, it took 20–25 minutes to complete the form. Respondents signed informed consent forms before completing the survey. The total final number of respondents was 1,802.

### Conceptual model

The HBM was employed as a primary reference to measure perception and preventive behavior on COVID-19. The HBM considers several main concepts that predict why people will take action to prevent, including individual characteristics, perceived susceptibility, severity, benefit, and self-efficacy. ^13^ This study consisted of 45 observed variables and six latent variables.

### Individual characteristics and community perception of COVID-19

Individual characteristics were represented by residence (R), age (A), gender (G), educational level (E), and occupation (O). Perceived susceptibility, severity, benefit, barriers, and self-efficacy are latent variables in this study. All questions in in the questionnaire in each latent variable were answered using a five-point Likert scale: 1 (strongly disagree), 2 (disagree), 3 (neutral), 4 (agree), and 5 (strongly agree). Observed variables X1-14, S1-5, BEN1-5, BAR1-5, and SE1-5 were used to measure perceived susceptibility, severity, benefit, barriers, and self-efficacy, in that order.

### Community preventive behavior against COVID-19 transmission

**Figure 1.**
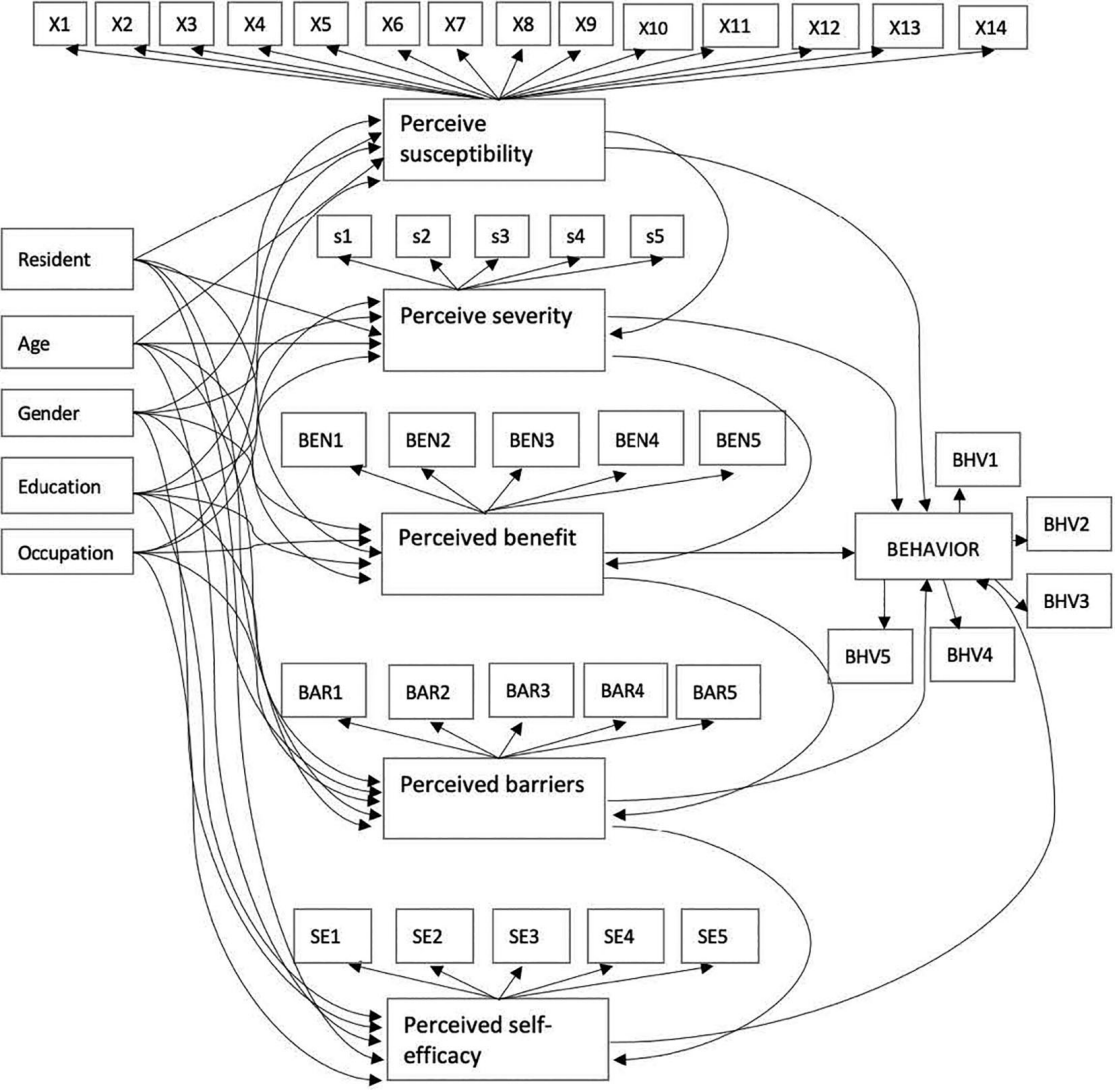
Proposed structural model of community preventive behavior against COVID-19. ^14^

Community preventive behavior was represented by six indicators in Figure 1 which include frequency of hosting (BHV1), frequency of visiting others (BHV2), frequency of work/study from office/school (BHV3), frequency of handshaking (BHV4), frequency travelling to a red zone (BHV5), and frequency of leaving the house when you are not feeling well (BHV6).

### Data analysis

Structural equation modelling (SEM) is a multivariate statistical analysis technique that is used to analyze structural relationships. This technique is the combination of factor analysis and multiple regression analysis, and it is used to analyze the structural relationship between measured variables and latent constructs. The authors used Lisrel version 8.8 software to construct the covariance-based SEM. SEM analysis can also be done using R. Steps of doing SEM analysis using R as follows ^15^:

a. Draw model
b. Input data in the form of covariance or correlation matrix
c. Identify the model
d. Assess parameter estimates
e. Assess fit measure (chi-square, degree of freedom, residual matrix, GFI, RMSEA)
f. Check the modification indices
g. Rerun the model till we get the best fit of the data to the model ad theory.

The six latent variables were perceived susceptibility, severity, benefit, barriers, self-efficacy, and preventive behavior. The 45 observed variables were presented in Table 1 include construct that build by all the observed variables.

**Table 1.** The observed variables.

No	Item	Scale	Description	Construct
1	R	1. City 2. Village 3. Housing area 4. Apartment	Residence	-
2	A		Age	-
3	G	1. Male 2. Female	Gender	-
4	E	1. No education 2. Elementary school 3. Junior high school 4. Senior high school 5. University	Education level	-
5	O	1. Unemployment 2. Labourer/employee 3. Student/college 4. Housewife 5. Entrepreneur 6. Pensionary 7. Others	Occupation	-
6	X1	1-5 Likert strongly disagree – strongly agree	I am at risk when not keeping a distance of at least 1-2 metres when in the work area.	Perceive Susceptibility
7	X2		I am at risk when not keeping a distance of at least 1-2 metres when at school.	
8	X3		I am at risk of not keeping a distance of at least 1-2 metres when at the mall/supermarket.	
9	X4		I am at risk when not keeping a distance of at least 1-2 metres when at the traditional market.	
10	X5		I am at risk when not keeping a distance of at least 1-2 metres at a restaurant/coffee shop/café.	
11	X6		I am at risk when not keeping a distance of at least 1-2 metres when at a touristdestination.	
12	X7		I am at risk when not keeping a distance of at least 1-2 metres when at a wedding reception.	
13	X8		I have a low risk of getting COVID-19 if I avoid using a bus/taxi.	
14	X9		I have a low risk of getting COVID-19 if I avoid using a commuter line.	
15	X10		I have a low risk of getting COVID-19 if I avoid using a small bus.	
16	X11		I have a low risk of getting COVID-19 if I avoid using motorcycle taxis/online.	
17	X12		I have a low risk of getting COVID-19 if I practice work/study from home.	
18	X13		I have a low risk of getting COVID-19 if I practice avoiding handshaking.	
19	X14		I have a low risk of getting COVID-19 if I practice avoiding travel to a red zone (an area with a high positive rate).	
20	S1		I may get COVID-19 and cause health impacts if I do not keep a distance ofat least 1-2 metresina public area.	Perceive severity
21	S2		I may get COVID-19 and cause health impacts if I do not avoid using public transportation.	
22	S3		I may get COVID-19 and cause health impacts if I do not work/study from home.	
23	S4		I may get COVID-19 and cause health impacts if I do not avoid handshaking.	
24	S5		I may get COVID-19 and cause health impacts if I do not avoid travel to a red zone.	
25	BEN1		I feel safe from COVID-19 if I keep a distance of at least 1-2 metresin a public area.	Perceive benefit
26	BEN2		I feel safe from COVID-19 if I avoid using public transportation.	
27	BEN3		I feel safe from COVID-19 if I work/study from home.	
28	BEN4		I feel safe from COVID-19 if I avoid handshaking.	
29	BEN5		I feel safe from COVID-19 if I avoid travel to a red zone.	
30	BAR1		I find it difficult to stayat least 1-2 metres from people in a public area.	Perceive barrier
31	BAR2		I find it difficult to avoid using public transportation.	
32	BAR3		I find it difficult to work/study from home.	
33	BAR4		I find it difficult to avoid handshaking.	
34	BAR5		I find it difficult to avoid travel to a red zone.	
35	SE1		It is easy for me to avoid travel to a red zone.	Perceive self-efficacy
36	SE2		It is easy for me to avoid handshaking.	
37	SE3		It is easy for me to work/study from home.	
38	SE4		It is easy for me to avoid using public transportation.	
39	SE5		It is easy for me to keep a distance of at least 1-2 metres when in a public area.	
40	BHV1		Frequency of hosting.	Behaviour
41	BHV2		Frequency of visiting others.	
42	BHV3		Frequency of work/study from office/school.	
43	BHV4		Frequency of handshaking.	
44	BHV5		Frequency travelling to a red zone.	
45	BHV6		Frequency of leaving the house when you are not feeling well.	

Descriptive statistics were presented as numbers and percentages for individual characteristics, and bar charts for perceptions and behavior. Descriptive statistical analysis was performed using IBM Statistical Package for the Social Sciences (SPSS) version 27 and presented using Microsoft Excel. The descriptive analysis simply can also be completed using Microsoft Excel. The SEM analysis was carried out using the following steps:

1) Identifying the degree of freedom of the structural model.
2) Assessing construct validity.
3) Assessing construct reliability.
4) Assessing structural model validity. ^16^^,^^17^

Perceived was categorized based on the total score. Good/high perceived were those who chose to “strongly agree” on every question. Regarding behavior, good behavior occurs if the respondent states never or rarely. Rarely was included in the category based on the assumption that people might be challenged to practice preventive behavior related to other factors that require them to leave, such as the environment or critical social activities that cannot be abandoned.

## Results

The study results are presented in several parts sequentially, starting with the respondent’s characteristics, followed by the descriptive statistics of the independent variable (community perception of the level of severity of COVID-19 disease), the descriptive statistics of the dependent variable (composite variable of community behavior), and the SEM analysis results.

Most of the respondents lived in the city (46.1%) and a housing area (36.1%), and only a few lived in villages (16.6%). A large majority of the respondents were women (74.5%), and approximately 80.0% were students and workers ( Table 2).

**Table 2.** Respondents’ characteristics.

	Frequency	%
Resident		
City	831	46.1
Village	299	16.6
Housing area	651	36.1
Apartment	21	1.2
Sex		
Male	460	25.5
Female	1342	74.5
Education Level		
No education	3	0.2
Elementary school	3	0,2
Junior high school	25	1.4
Senior high school	885	49.1
University	886	49.2
Occupation		
Unemployment	128	7.1
Labourer/employee	552	30.6
Student/college	958	53.2
Housewife	35	1.9
Entrepreneur	106	5.9
Pensionary	3	0.2
Others	20	1.1

In terms of perceived susceptibility ( Table 3), more than half of respondents (>50%) chose to strongly agree to practice recommended behaviors such as social distancing at least 1-2 meters in public areas; almost half of them strongly agree that they had a low risk of getting COVID-19 when avoiding using public transportation, practicing work/study from home, handshaking, and travelling to a red zone. Most respondents also strongly agree that if they are exposed to COVID-19, it will affect their health if they do not practice recommended behaviors. Likewise, concerning perceived benefit, more than 50% of respondents strongly agree that they can avoid getting COVID-19 if they practice these behaviors. In terms of perceived barriers, less than 20% felt that it would be challenging to implement the recommended behaviors. Regarding self-efficacy, this is defined as the conviction that one can successfully practice a certain behavior, ^13^ the survey showed that less than 50% were confident that they could implement the recommended behavior.

**Table 3.** Distribution (%) of perceived items (N=1802).

Perceive	1 (%)	2 (%)	3 (%)	4 (%)	5 (%)
**Perceive Susceptibility**					
***I am at high risk when I am not keeping a distance of at least 1-2 m when in apublic area***					
Working area	3.5	2.1	6.3	27	61.2
School	3.3	2.6	8.0	28	58.1
Mall/supermarket	3.1	1.7	5.7	22.6	67
Traditional market	2.9	1.6	5.5	20.5	69.5
Restaurant/coffee shop/café	2.7	2.6	7.5	27.1	60
Tourism objects	3.4	3.2	10.5	27.1	55.8
Wedding reception	2.9	1.8	6.4	22.1	66.8
***I have a low risk of getting COVID-19 if I avoid using public transportation***					
Bus/taxi	6	6	11.4	25.9	50.8
Commuter line	6.4	5.6	12.2	23.9	51.9
City transport	6.4	5.1	9.5	23.5	55.5
Offline motorcycle taxi/online	5.1	10.8	28.4	30.7	25
***I have a low risk of getting COVID-19 if I practice following behaviours***					
Work/study from home	3.4	0.9	5.2	19.3	71.2
Avoid handshaking	2.7	0.9	3.4	19	74
Avoid travel to red zone (are with high positive rate)	2.6	1	3.3	16.6	76.5
**Perceive Severity**					
***I may get COVID-19 and cause health impactstomyself if I do notfollow behaviours***					
Keep distance at least 1-2 metre when at public area	3.4	1.6	4.9	26.2	63.9
Avoid using public transportation	3.8	2.9	13.5	29.3	50.4
Work/study from home	5.0	2.4	7.3	27.9	57.4
Avoid handshaking	4.3	1.9	4.2	24.5	65.1
Avoid travel to red zone (are with high positive rate)	4.6	1.6	4.2	21.0	68.6
**Perceive Benefit**					
***I feel safe and get off COVID-19 if I practice following behaviours***					
Keep distance at least 1-2 metre when at public area	2.6	1.1	4.8	23	68.5
Avoid using public transportation	2.3	1.6	9.3	23.9	62.8
Work/study from home	2.6	1.3	5.9	22.4	67.8
Avoid handshaking	2.6	1.2	4.6	20	71.8
Avoid travel to red zone (are with high positive rate)	2.5	1.2	4.3	18.2	73.9
**Perceive of Barrier**					
***I find it difficult to practice the following behaviours***					
Keep distance at least 1-2 metre when at public area	23.3	24.4	16.4	23.6	12.4
Avoid using public transportation	34.8	25.2	15.8	14.1	10
Work/study from home	35.2	25.1	15.9	12.4	11.4
Avoid handshaking	38.8	28.9	14.2	10.2	7.9
Avoid travel to red zone (are with high positive rate)	37.0	25.6	15.9	12.2	9.3
**Self-Efficacy**					
***It is easy for me to practice following behaviour***					
Keep distance at least 1-2 metre when at public area	4.5	11.8	17.0	30	36.7
Avoid using public transportation	4.3	9.8	15.5	23.8	46.5
Work/study from home	6.3	8.0	13.0	24.6	48
Avoid handshaking	3.1	5.0	11.2	28.4	52.4
Avoid travel to red zone (are with high positive rate)	5.0	7.2	13.0	23.9	50.9

Notes: 1: strongly disagree; 2: disagree; 3: neutral, 4: agree, 5: strongly agree.

For each type of recommended behavior as mentioned in Table 1 to prevent the transmission of COVID-19, more than 50% of respondents never and rarely hosted, visited others, worked from the office/school, shook hands, travelled to a red zone, and left the house when not feeling well ( Figure 1).

Based on the composite of each perceived item, it was found that only 16% of the respondents had a high percentage of good perceived susceptibility; perceived severity was 43%, perceived benefit was 54%, perceived barrier was 3%, self-efficacy was 19%, and only 60% of respondents practiced good behaviours.

## SEM results

### Identify degree of freedom of structural model

The second step in SEM analysis is to run the identification of observed variables. A general requirement for identifying any type of model in SEM are the model’s degrees of freedom which must be a least zero (df _M_ ≥ 0). Hence, the solution to meet the requirement is to identify whether the model is under identified, just identified, or overidentified. Overidentified is mandatory in order to meet the requirement. An overidentified structural equation model is identified and has more observations than free parameters (df _M_>0)(2). In this study, we found the degree of freedom value to be 821, hence it was concluded that the model was over-identified. Thus, the next step of the analysis can be completed.

### Construct validity

Construct validity was performed to test whether the instrument or measurement variable could describe the latent variable correctly and precisely. For this, two tests were conducted: validity, which consisted of convergent and discriminant validity, and reliability. ^14^ A convergent validity test examined the loading factor value of the measurement variable in each latent variable construct. If the loading factor value was greater than 0.50, the latent variable construct had good convergent validity. ^14^ The results of the convergent validity test showed that almost all items in this study had a loading factor value of more than 0.5, except for items X11 and BHV3. These two items have a loading factor of ≤ 0.5, which indicates that they do not meet the criteria for convergent validity. Hair *et al.* (2019) stated that items with low loading factors that do not meet the limits of convergent validity should be excluded from the measurement of latent variables. Therefore, items X11 and BHV3 in this study were not included in the measurement of the latent variable. The convergent validity test was then carried out for a second time. All of the items had good convergent validity, which was shown by loading factor values of more than 0.5. The discriminant validity test was carried out by comparing the root value of each latent variable’s average variance extracted (AVE) with the correlation of these latent variables with other latent variables. If the root value of the variable AVE was greater than the correlation of the variable with other variables in the model, the indicator/question item had good discriminant validity.

Table 4 shows the AVE root value for each latent variable and the correlation coefficient between the latent variables. The value of the AVE root is shown as the value on the diagonal of the matrix, while the values beside and below the AVE root are the correlation coefficients between two pairs of variables. The results of the evaluation of discriminant validity show that the root value of AVE in each latent variable is greater than the correlation coefficient of the latent variable with other latent variables in the structural model. Thus, it can be stated that the items/instruments in this study have good discriminant validity.

**Table 4.** Results of the discriminant validity test.

	Suscept	Severity	Benefit	Barrier	Efficacy	Behaviour
Suscept	**0.822**	-	-	-	-	-
Severity	0.730	**0.911**	-	-	-	-
Benefit	0.590	0.810	**0.936**	-	-	-
Barrier	-0.160	-0.210	-0.260	**0.737**		
Efficacy	0.080	0.100	0.120	-0.480	**0.725**	
Behaviour	-0.120	-0.150	-0.160	0.460	-0.320	**0.630**

### Construct reliability

The construct reliability test was done by examining the composite reliability value. If the combined reliability value is greater than 0.7, it can be said that the variables in the study already have reliable indicators/question items. ^16^ All latent variables were found to have a composite reliability value of more than 0.7, which means that each variable has a consistent measurement indicator and good internal consistency.

## Structural model validity

Two analyses were conducted to evaluate the structural model validity:1) dependency test and 2) assessing the goodness-of-fit of the model.

### Dependency test

The dependence relationship test was employed by looking at the path coefficient and its *p*-value in the structural model. The path coefficient shows the magnitude and direction of the relationship between the two variables. According to Table 5, sex had a statistically significant relationship with perceive susceptibility; perceive susceptibility had a statistically significant relationship with perceive severity; age, sex, and education had significant relationships with perceive benefit; perceive benefit, resident, and sex had a significant relationship with perceive barrier; and perceive barrier and occupancy had a significant relationship with perceive self-efficacy. Regarding behavior, only perceived barriers and self-efficacy had statistically significant relationships with COVID-19 prevention behavior. The final structural model is as follows: behaviour = - 0.018*susceptibility - 0.041*severity - 0.00045*benefit + 0.39*barrier - 0.13*self-efficacy.

**Table 5.** Results of the SEM statistical test.

Latent variable	Independent	Path coefficient	Std. error	t-Statistics	p-value
Perceived Susceptibility	Resident	0.009	-0.010	0.870	0.273
	Age	0.005	-0.005	1.020	0.237
	Sex	0.180	-0.024	7.540	0.000
	Education	0.005	-0.006	0.860	0.276
	Occupancy	0.045	-0.029	1.520	0.126
Perceived Severity	Perceived Susceptibility	0.720	-0.020	35.810	0.000
	Regional	0.012	-0.007	1.690	0.096
	Age	0.001	-0.003	0.280	0.384
	Sex	0.014	-0.017	0.810	0.287
	Education	-0.008	-0.005	-1.680	0.097
	Occupancy	-0.020	-0.021	-0.960	0.252
Perceived Benefit	Perceived Severity	0.810	-0.018	44.660	0.000
	Resident	-0.010	-0.006	-1.550	0.120
	Age	-0.009	-0.003	-3.030	0.004
	Sex	0.034	-0.015	2.270	0.030
	Education	0.008	-0.004	2.040	0.050
	Occupancy	0.032	-0.018	1.770	0.083
Perceived Barrier	Perceived Benefit	-0.240	-0.026	-9.260	0.000
	Resident	-0.028	-0.010	-2.770	0.009
	Age	-0.009	-0.005	-1.940	0.061
	Sex	-0.130	-0.025	-5.050	0.000
	Education	0.009	-0.007	1.340	0.163
	Occupancy	0.051	-0.031	1.670	0.099
Perceived Self-Efficacy	Perceived Barrier	-0.480	-0.031	-15.860	0.000
	Resident	0.008	-0.010	0.840	0.280
	Age	-0.003	-0.005	-0.550	0.343
	Sex	-0.022	-0.024	-0.940	0.256
	Education	0.001	-0.006	0.081	0.398
	Occupancy	0.063	-0.029	2.150	0.040
Individual Behaviour	Perceived Susceptibility	-0.018	-0.037	-0.500	0.352
	Perceived Severity	-0.041	-0.053	-0.780	0.294
	Perceived Benefit	0.000	-0.045	-0.010	0.399
	Perceived Barrier	0.390	-0.036	10.930	0.000
	Perceived Self-Efficacy	-0.130	-0.032	-4.000	0.000

The goodness-of-fit model assessment results in Table 6. showed that none met the goodness-of-fit criteria. Therefore, the structural model in this study was not a fit for the data and was not a fit for describing the empirical phenomenon under study.

**Table 6.** Goodness-of-fit index results.

Goodness-of-fit index	Cut of value	Value	Conclusion
Probability of χ ^2^	>0.050	0.000	No fit
RMSEA	<0.080	0.140	No fit
GFI	>0.950	0.560	No fit
AGFI	>0.950	0.490	No fit
SRMR	<0.100	0.140	No fit
CFI	>0.950	0.850	No fit
NFI	>0.950	0.850	No fit
PNFI	>0.950	0.770	No fit
PGFI	>0.950	0.480	No fit
IFI	>0.950	0.850	No fit
RFI	>0.950	0.830	No fit

Notes: RMSEA=Root Mean Square Error of Approximation; GFI=Goods of Fit Index; AGFI=Adjusted Goodness of Fit Index; SRMR=Standardized Root Mean Square; CFI=Comparative Fit Index; NFI=Normed Fit Index; PNFI=Parsimonious Normed Fit Index; PGFI=Parsimonious Goods of Fit Index; IFI=Incremental Fit Index; RFI=Relative Fir Index.

## Discussion

The results of grouping all perceived items showed that only a few respondents (16%) had beliefs about the chances of experiencing COVID-19 (perceived susceptibility), but 43% of respondents believed how serious the COVID-19 effects were on their health. More than half of the respondents believed that the recommended behaviors to prevent COVID-19 infection could protect them from getting an infection (perceived benefit). Only 3% of respondents believed some things hindered the practice of recommended behaviors (perceive barrier), and only 19% believed that they could practice the recommended behaviors (perceive self-efficacy). This study’s results are similar to those conducted in India, Sri Lanka, Iran, and Ethiopia. ^8^^,^^10^^,^^18^^,^^19^ The HBM theory holds that people are likely to practice preventive behaviors or actions if: 1) They believe that it will reduce their risks, ^13^ 2) They believe themselves to be susceptible to COVID-19 infection, 3) They believe that this condition would have a potentially serious impact, 4) They believe that recommended actions/behaviours would be beneficial in reducing their susceptibility to or severity of the virus infection, and 5) They believe that the anticipated benefits of doing preventive behaviours/actions outweigh the barriers.

Perceived susceptibility was not a significant predictor of behavior in this study. This finding is consistent with studies that measured adherence to COVID-19 precautionary measures in China ^20^ and Korea. ^21^^,^^22^ A study that used a HBM framework to look at how Saudi Arabian students at Jazan University felt about the COVID-19 vaccine found that perceived susceptibility was not a good predictor of how they felt about getting the vaccine. ^23^ However, a similar study conducted in Malaysia found that high perceived susceptibility to COVID-19 infection was also associated with the behavior of vaccination intention. ^24^ A study that measured student behavior in the US related to non-pharmaceutical interventions (hand washing with soap and water, use of hand sanitizer, wearing a face mask in public, and practicing social distancing) found that perceived susceptibility was associated with multiple interventions more frequently. ^25^

A previous study conducted by Du Min e *t al.* found that low perceived risk was associated with vaccine hesitancy. ^26^ As with perceived susceptibility, perceived severity was not a significant predictor of preventive behavior in this study. Several studies regarding behavior change using BHM had similar results. ^22^^,^^24^^,^^25^ Perceived severity, on the other hand, was a significant predictor of preventive behaviors. ^8^^,^^20^^–^^23^^,^^27^ Perceived benefit is one predictor that is not a significant predictor of preventive behavior. This finding is inconsistent with several studies that used BHM to predict behavior change, particularly in relation to COVID-19 prevention. ^8^^,^^20^^,^^21^^,^^23^^–^^27^

This study revealed that the perceived barrier was a significant predictor of COVID-19 preventive behaviour. This result is different from a behaviour study conducted in Sri Lanka and Iran, which found that perceived benefit and self-efficacy had a significant positive relationship to COVID-19 prevention behaviour. ^10^^,^^28^ However, these results were similar to a study conducted in Ethiopia that measured student eating behaviour using the HBM theory in the US ^29^ and other behavioural studies in Iran, India, and Hong Kong. ^8^^,^^19^^,^^30^ This study also found that perceived self-efficacy was a significant predictor of COVID-19 preventive behaviour. The results show that, with lower self-efficacy, people were likely to practice COVID-19 prevention behaviour. This result is similar to several studies that examined behaviours using the BHM theory. Those studies found that perceived self-efficacy has a significant relationship to behaviour. ^10^^,^^18^^,^^19^^,^^21^^,^^28^^,^^31^ In contrast, a study that assessed the student’s behaviour on the non-pharmaceutical intervention of COVID-19 found that perceived self-efficacy was not a significant predictor of behaviour change. In theory, an individual with good self-efficacy tends to practice recommended action/behaviour, ^13^ which is the preventive behaviour of COVID-19. However, this study was unable to confirm these findings.

The findings in this study illustrated that most respondents (97%) had no barriers to practising the recommended behaviour. Still most respondents (81%) were not confident that they could fully implement the recommended prevention behaviours. As many as 60% of respondents practised COVID-19 prevention behaviour well. In knowledge attitude practice (KAP) studies conducted in Indonesia, this finding (percentage of good behaviour) tended to be lower than the other two findings in Indonesia, where the rate of those who performed the correct behaviour for the prevention of COVID-19 was more than 90%. ^32^^,^^33^ Studies conducted in other countries also found that respondents who practised COVID-19 prevention behaviours were relatively high (>70%), such as in China, Nepal, Malaysia, Vietnam, and India. ^34^^–^^38^

Fundamentally, perceived susceptibility and severity affect how a person decides to act. ^13^ However, most respondents (84%) had low perceived susceptibility in this study. This means that most respondents did not believe that they were also at risk of being affected by COVID-19. This perception represented an obstacle for someone to implement recommended behaviour. It was also known that only 43% believed that if they were infected with COVID-19, they would experience harmful consequences for their bodies. Only half of the respondents believed that the recommended behaviour was able to protect them from COVID-19. This might relate to the information they obtained day-to-day. It was possible that most of them did not have a clear idea about the pathophysiology and epidemiology of COVID-19, made worse by unreliable news or hoaxes circulated on social media, which may have increased negative perceptions about COVID-19. ^8^^,^^39^

Regarding obstacles to implementing the recommended behaviour, a few respondents said it was extremely difficult to implement the behaviours (3%). This overall perception then leads to a low belief that respondents are able to implement the recommended behaviour. As a consequence, it will affect COVID-19 prevention behaviour practice. Perception is theoretically influenced by many factors, including demographics and level of knowledge. ^8^^,^^10^^,^^13^^,^^18^^,^^32^^,^^33^ A study conducted in China found that knowledge was influenced by educational level and domicile. ^34^ Good knowledge can form a good attitude, which then creates a good perceived. ^40^^,^^41^ COVID-19 was a pandemic that touched almost every facet of human existence. People had to adjust their daily routines to accommodate local government rules in order to reduce the virus transmission, and these behavioural shifts may last long after the disease has passed. ^42^ Increasing the respondents’ knowledge is essential to narrowing the gap between knowledge and practice, including myths, hoaxes circulating about COVID-19, and facts. Several important factors that may affect perceived self-efficacy are related to social norms and trust ^43^ in the community. Unfortunately, neither of these factors (including knowledge) was investigated in this study.

The structural model of COVID-19 prevention behaviour in this study was not intended to describe the empirical phenomenon of preventive behaviour. Byrne stated that, if possible, researchers are advised to modify the model by using modification indices (MI) in SEM testing. He said that models with an MI score of more than 10 deserve attention for modification. ^44^ Already it has proved that to confront the increase in demand for care, the need for long-term care workforce, and the costs associated with care. ^45^ However, Hair *et al.* suggested that modifying the model should not change the model’s structure significantly. ^16^

This several studies found a gap in knowledge to inform changes in policies on infection-prevention measures in the community, community infection procedures, the frequency of testing, etc. ^8^^,^^39^^,^^46^ The unreliable information may increase the negative perception of COVID-19, leading to the community’s obedience to suggested preventive behaviors. ^39^ Moreover, people are more worried about their families and economic conditions due to the spreading pandemic than about complying with lockdown or restriction policies. Several factors that might be related were not examined in this study, such as level of knowledge, social norms, or trust.

### Study limitations

Due to the social restrictions (the outdoor community activities are limited by the government based on the law announced every two weeks except for emergency purposes) in Indonesia, the data collection was conducted using a Google form without using a selected sampling method. Thus, the total number of respondents in this study was not representative of the total population in Indonesia and the entire territory of Indonesia. In addition, the entire population of Indonesia did not have the same opportunity to be selected as respondents due to limitations related to internet coverage and utilities. Since the prevalence and impact of COVID-19 are changing dramatically, across-sectional studies were not the best method for describing the changes. Better results might be possible using a longitudinal study.

## Conclusions

This study found that more than half of the respondents still had low perceived susceptibility and severity, but more than half had high perceived benefits. Only a few respondents had significant barriers to implementing COVID-19 transmission prevention behaviors. Still, most respondents had low perceived self-efficacy, and only 60% had good behaviors related to COVID-19 prevention.

The results of this study can assist policymakers in developing health interventions related to the preventive behavior of COVID-19 transmission in the community. We recommend increasing perceived susceptibility and severity by providing the correct information about COVID-19 in the local cultural context. The results of this study can also be used as a reference for evidence-based policies so that the government can prioritize the types of interventions to improve public understanding and compliance to prevent the transmission of COVID-19.

The findings of similar studies conducted in other countries showed some consistency. By improving perceived susceptibility and severity, there would be an increase in respondents’ knowledge, increasing perceived susceptibility and severity. The results of this study can, not only be used for COVID-19 prevention policies but can also be used as a reference in making prevention policies against many types of diseases that require community behavior changes. Therefore, when making policies, there are stages that must first be met so that behavior can change, as described in the results of this study.

## Ethics approval and consent to participate

The Commission approved this study for Research Ethics and Public Health Service, Faculty of Public Health, University of Indonesia Number: Ket-436/UN2.F10. D11/PPM.00.02/2021.

## Consent for publication

Informed consent was obtained from all subjects involved in the study.

## Author contributions

All authors made substantial contributions to this research and approved the final manuscript. TE and TS contributed to every step of the study (research concept, design, data interpretation, writing, and review). SP contributed to the research review.

## Data Availability

Dyrad. Data for: Community perception and COVID-19 prevention, https://doi.org/10.5061/dryad.pnvx0k6rp. ^47^ This project contains the following underlying data:
•
DATA_FINAL_tio-edit2.csv (Data include all variables in the questionnaire. The data contain 1802 respondents and 89 variables. The variable names of questions 1–23 were given according to the keyword in each question, while the variable names for question number 24–35 were specified according to the question’s number and its answer option. It is recommended to read the variable code definition in sheet 2 and the area code in sheet 3.) Data are available under the terms of the Creative Commons Zero “No rights reserved” data waiver (CC0 1.0 Public domain dedication). Figshare. IMPLEMENTATION OF SOCIAL POLICY DISTANCING IN EFFORT PREVENTION COVID-19 IN INDONESIA. https://protect-us.mimecast.com/s/qsa7CkRwomfYPpGvjC2JN1U?domain=doi.org ^14^ This project contains the following supplementary material:
• Kuesioner Penelitian.pdf (the google questionnaire used in this study), https://doi.org/10.6084/m9.figshare.23292686.v2. ^48^ Data are available under the terms of the Creative Commons Attribution 4.0 International license (CC-BY 4.0).
