# Supplementary material for: Community preventive behaviour and perception on the severity of COVID-19 disease in Indonesia, 2021-2022: Structural equation modelling
Source: F1000Res. 2024 Oct 3;12:966. Originally published 2023 Aug 10. [Version 3] doi: 10.12688/f1000research.135262.3 (PMC11377921; doi:10.12688/f1000research.135262.3)
Supplement: Peer review history

# Reviewer report: Reviewer response for version 3

**DOI:** 10.5256/f1000research.172626.r328969

**Published:** 2024-10-21

**Authors:** Husnah Husnah

**Affiliations:** 1 Department of Nutrition, School of Medicine, Syiah Kuala University, Banda Aceh, Aceh, Indonesia

Community preventive behaviour and perception on the severity of COVID-19 disease in Indonesia, 2021-2022: Structural equation modelling [version 3; peer review: 1 approved, 2 approved with reservations]

 Based on the latest revision that I have studied, there are several suggestions for improvement.

**A. Background**

 The background and methods are appropriate

**B. Results**

 1. The results of the research need to be supplemented with data about the number of respondents written down, most of which should be called numbers, not just percentages. Example: More than halfof respondents should write 901 (50%) instead of 1802

 2. The value of a significant relationship should be written down, not just *p*<0.05

**C. Conclusion**

 In the sentence respondents have a low perception of vulnerability (84%) and severity (67%), it is better to add the number of respondents, then write the percentage

Is the work clearly and accurately presented and does it cite the current literature?

Yes

If applicable, is the statistical analysis and its interpretation appropriate?

Partly

Are all the source data underlying the results available to ensure full reproducibility?

Yes

Is the study design appropriate and is the work technically sound?

Yes

Are the conclusions drawn adequately supported by the results?

Partly

Are sufficient details of methods and analysis provided to allow replication by others?

Yes

Reviewer Expertise:

Nutrition Department

I confirm that I have read this submission and believe that I have an appropriate level of expertise to confirm that it is of an acceptable scientific standard, however I have significant reservations, as outlined above.

# Reviewer report: Reviewer response for version 2

**DOI:** 10.5256/f1000research.159876.r233985

**Published:** 2024-09-05

**Authors:** Rotimi Oguntayo

**Affiliations:** 1 Department of Psychology, Universidad Autonoma de Ciudad Juarez, Ciudad Juarez, Chihuahua, Mexico

Dear Authors!

 Thank you for attending to all my questions, and the amendment made to the article is commendable. At this point, I recommend that this work be indexed.

Is the work clearly and accurately presented and does it cite the current literature?

Partly

If applicable, is the statistical analysis and its interpretation appropriate?

Yes

Are all the source data underlying the results available to ensure full reproducibility?

Yes

Is the study design appropriate and is the work technically sound?

No

Are the conclusions drawn adequately supported by the results?

Partly

Are sufficient details of methods and analysis provided to allow replication by others?

Partly

Reviewer Expertise:

Psychology (Crises Prevention and management)

I confirm that I have read this submission and believe that I have an appropriate level of expertise to confirm that it is of an acceptable scientific standard.

# Reviewer report: Reviewer response for version 2

**DOI:** 10.5256/f1000research.159876.r296323

**Published:** 2024-07-24

**Authors:** Valentina Lucia La Rosa

**Affiliations:** 1 Department of Educational Sciences, University of Catania, Catania, Italy

The manuscript “Community Preventive Behaviour and Perception on the Severity of COVID-19 Disease in Indonesia, 2021-2022: Structural Equation Modelling" provides a valuable contribution to the understanding of COVID-19 perceptions and preventive behaviors in Indonesia. The use of HBM and SEM provides a robust framework for their analysis. However, addressing the revisions outlined above will significantly strengthen the manuscript and improve its suitability for indexed.

1. The Authors should provide a broader theoretical basis for their SEM analysis. The HBM is a well-established model, but the authors need to explicitly link their research questions and hypotheses to the specific constructs of the HBM. In addition, they should discuss how the HBM influenced their choice of variables and the relationships tested in their SEM model.
2. The Authors need to provide more details about their data collection methods. In particular, they should address how they ensured that respondents did not complete the Google form multiple times and how they ensured an even distribution of respondents across the 38 provinces of Indonesia. If these measures were not taken, this point should be acknowledged as a limitation of the study.
3. The goodness-of-fit indices suggest that the proposed model does not adequately fit the data. The authors should consider revising the model by exploring alternative constructs or pathways that may better represent the empirical data.
4. The Authors are required to provide a detailed explanation of the steps taken to address issues with the model fit and any changes that were made to improve the model.
5. The Authors should expand the discussion of the implications of their findings, with particular focus on the non significant predictors, and discuss how these findings contribute to our understanding of preventive behaviors in the context of COVID-19.
6. I suggest including references to studies that have examined the perceived health risk associated with COVID-19 in different populations, such as adolescents and college students, to provide a broader context and comparison for your findings. Some of these studies include: 10.3389/fpsyg.2020.559951; 10.3390/ejihpe13080108.
7. The manuscript should be thoroughly proofread to correct grammatical errors and improve its readability.

Is the work clearly and accurately presented and does it cite the current literature?

Partly

If applicable, is the statistical analysis and its interpretation appropriate?

Partly

Are all the source data underlying the results available to ensure full reproducibility?

Partly

Is the study design appropriate and is the work technically sound?

Yes

Are the conclusions drawn adequately supported by the results?

Partly

Are sufficient details of methods and analysis provided to allow replication by others?

Partly

Reviewer Expertise:

Development in the life span and the impact of critical events such as the COVID-19 pandemic

I confirm that I have read this submission and believe that I have an appropriate level of expertise to confirm that it is of an acceptable scientific standard, however I have significant reservations, as outlined above.

## References

[1] : Adolescents in Quarantine During COVID-19 Pandemic in Italy: Perceived Health Risk, Beliefs, Psychological Experiences and Expectations for the Future. *Front Psychol* .2020;11: 10.3389/fpsyg.2020.559951 559951 10.3389/fpsyg.2020.559951 33071884 PMC7538632

[2] : University Experience during the First Two Waves of COVID-19: Students' Experiences and Psychological Wellbeing. *Eur J Investig Health Psychol Educ* .2023;13(8) : 10.3390/ejihpe13080108 1477-1490 10.3390/ejihpe13080108 37623305 PMC10453088

# Response

**Published:** 2024-09-27

**Authors:** Tris Eryando

**Affiliations:** Biostatistics and Population, Universitas Indonesia, Depok, West Java, Indonesia

1. Response to no. 1: We propose a comprehensive approach to understanding behavior change using the Health Belief Model (HBM), as opposed to focusing to understand behaviour change partially. The HBM offers a holistic perspective on behaviour change processes. However, when utilizing HBM as a theoretical framework, there were latent variables that did not directly observed. These latent variables are represented by various indicators/observe variable. Consequently, Structural Equation Modelling (SEM) is the most suitable analytical method for examining relationships between variables (latent variable and measurement/indicator) within the HBM framework, as it allows for the analysis of both latent variables and the overall model as a single, cohesive unit. We added the additional statement in the article.
2. Response to no. 2: We did not restrict respondents to just entry the form one time. However, once the data collected and stored in to the database, researcher did data checking particularly to identify double/triple number. The researcher selected one data randomly and deleted the rest with the same number. There was possibility that one person will entry multiple times using different identity. We added the additional statement in the article.
3. Response to no. 3: Unfortunately, we didn’t develop or explore the alternative construct after finding that the model did not fit the data since the aim of this study was to examine changes in community behaviour to prevent local COVID-19 transmissions and changes in community perceptions about the severity level of COVID-19. This study also intended to prove whether the construct proposed can predict the behaviour or not and the result showed that it could not. We did not re-construct the model considering Hair et al. suggestion that modifying the model should not change the model’s structure significantly.
4. Response to no. 4: In order to propose the good model, we have explained the one by one in the paper which include identifying degree of freedom, construct validity, discriminant validity, construct reliability, dependency and goodness of fit index. During the process, we excluded 2 variable that disturbed the data. However, when the model builds and tested with the goodness of fit index, we did not continue to re-structure the model. We refer to Hair et al. suggestion that modifying the model should not change the model’s structure significantly. We already stated it in the paper.
5. We added reference as suggested.

# Reviewer report: Reviewer response for version 1

**DOI:** 10.5256/f1000research.148373.r201070

**Published:** 2023-09-22

**Authors:** Rotimi Oguntayo

**Affiliations:** 1 Department of Psychology, Universidad Autonoma de Ciudad Juarez, Ciudad Juarez, Chihuahua, Mexico

**Abstract (Minor Revision):** In the conclusion section, significant results have been attached to preventive behavior and community perceptions of the severity of COVID-19 disease, but to support objective data, it is necessary to attach data in the form of accurate numbers in accordance with the findings in the field. In the results section, you mentioned that there is a relationship between self-efficacy and perceived influence on preventive behavior. How did you come to the conclusion that these variables are related?

**Methodology (Major Revision):** This is an interesting study because it is a large-scale study of the country of Indonesia. I have high hopes that this research will be successfully indexed so that it can be used as a reference by other studies in information about Community preventive behavior and perception of the severity of COVID-19 disease in Indonesia. As with other research articles published in various journals, your research article needs significant improvement to enhance its readability and credibility. You collected data through a Google Form distributed via various social media channels, resulting in thousands of responses. However, it is essential to address potential biases in data collection. How did you ensure that respondents didn't fill out the Google Form multiple times? Was there strict control over the form submissions? Additionally, you mentioned that respondents were spread across Indonesia. How did you ensure even distribution among Indonesia's 38 provinces? Please provide details or references for this distribution method. If you were unable to meet up with any of these criteria, list them as your study limitations.

**Study and Sample (Minor Revision):** This research aims to reach the Indonesian public, including contributions from social media influencers. To ensure the research's representativeness, please clarify how you ensured that the sample accurately represents all Indonesians. Your respondents are identified as Indonesian citizens, but how did you verify their citizenship? What measures were in place to prevent participation by non-Indonesian residents? Consider addressing these potential sources of bias. If you were unable to meet up with any of these criteria, list them as your study limitations.

**Conceptual Model (Major Revision):** This research employs the Health Belief Model (HBM) as the primary theoretical framework. It is crucial to emphasize that Structural Equation Modeling (SEM) relies on a well-established theory to validate models using real data. The direction of relationships in the model must align with theoretical support. Additionally, theoretical underpinnings are essential in determining the appropriate sample size for SEM and ensuring sample adequacy when testing a theory-based model. Please provide a more comprehensive, precise and concise theoretical foundation for your SEM analysis.

**Grammatical Errors (Minor Revision):** Correct the English writing errors in the article generally. Moreover, consider updating the COVID-19 incidence rate beyond the end of 2021, extending it into 2022, to enhance the study's accuracy and encompassing quality.

**Results:** The results section requires elaboration and clarification to explain how you arrived at the conclusion regarding the relationship between self-efficacy and perceived influence on preventive behavior for readers who not statistic expert to be able to read and understand while retaining the journal format.

**Discussion:** Expand on the implications of the results and how they relate to the theoretical framework. Discuss the broader significance of your findings in the context of COVID-19 prevention in Indonesia and HBM concept for clinical practice in local context. Also, consider inclusion of more recent similar COVID-19 incidence data in other country to buttress your results in this section.

**Conclusion with Limitations and Implications:** Summarize the key findings, discuss the study’s limitations as suggested above, and provide implications for clinical practice or public health initiatives, and future research to ascertain replicability of your work.

**Indexing of the Paper:** The indexing of the paper is contingent upon addressing the major revisions outlined in the methodology and conceptual model sections. Minor revisions should also be completed in the abstract, study and sample, grammatical errors, and the inclusion of more recent COVID-19 incidence data in the discussion section. Once these revisions are made, the paper will be reevaluated for indexing.

Is the work clearly and accurately presented and does it cite the current literature?

Partly

If applicable, is the statistical analysis and its interpretation appropriate?

Yes

Are all the source data underlying the results available to ensure full reproducibility?

Yes

Is the study design appropriate and is the work technically sound?

No

Are the conclusions drawn adequately supported by the results?

Partly

Are sufficient details of methods and analysis provided to allow replication by others?

Partly

Reviewer Expertise:

Psychology (Crises Prevention and management)

I confirm that I have read this submission and believe that I have an appropriate level of expertise to confirm that it is of an acceptable scientific standard, however I have significant reservations, as outlined above.

# Response

**Published:** 2023-11-16

**Authors:** Tris Eryando

**Affiliations:** Biostatistics and Population, Universitas Indonesia, Depok, West Java, Indonesia

Thank you for your valuable inputs to this paper.

 We already revised almost all part according to your input. Following are our response:

**Abstract**

 The abstract session will be revised according to the article’s revision. The number will be put accordingly. The conclusion of relationship between self- efficacy and perceived was made based on statistical test. It will be added in the abstract.

**Methodology**

 Due to the social restriction during the pandemic, the data was collected using google form. We did not do any specific treatment to limit the frequency of people submission as well as the respondents location. We also could not prevent respondent from other country to fill the link. However, when we did data cleaning, we erased data which is not located in Indonesia and having the same identity.

 Because of data in this study was collected through google link, the respondent indeed did not distributed evenly in all area in Indonesia and not to mention to represent and that is for sure could not represent all Indonesian population.

 Thus, this study is vulnerable to information and selection bias.

 All this information will be added in the article as our study limitation including our suggestion to minimize the bias for the further study.

**Study and sample**

 As we already wrote down in limitation part, sample in this study is not representing Indonesian population. We only distributed the link as much as we can and even engage with social media influencer to speed up the distribution. We wanted to collect as much as we can during the data collection time with the minimum sample size as our benchmark.

 As mentioned previously, we cannot control response from other country, as well as double respondent, however when we did data cleaning, we dropped out respondent who are not located in Indonesia and having the same identity.

 Indeed, this situation somehow my lead to the information and selection bias. Hence, we also provided our recommendation for further study. 

**Conceptual model**

 This study employed HBM theory as the conceptual framework and the data was collected according to each variable mentioned in the theory. The framework was described in the article both in Figure 1 and Table 1. The point of using SEM is to test a theory by specifying a model that represent predictions of that theory among plausible constructs measured with appropriate observed variables. In this study, we tested the HBM theory using the collected data. The output of this analysis is to identify whether the analysis deals with substantive theoretical issues regardless of whether or not a model is retained. Thus, in principle, the analysis in this study did not highlight/emphasize the statistical relationship among 2 variables, instead a model as a whole. We will add the explanation in the paper,

 Grammatical Error, Result, Discussion, and **Conclusion with Limitations and Implications >> we directly revised in the document**

# Reviewer report: Reviewer response for version 1

**DOI:** 10.5256/f1000research.148373.r195846

**Published:** 2023-09-13

**Authors:** Husnah Husnah

**Affiliations:** 1 Department of Nutrition, School of Medicine, Syiah Kuala University, Banda Aceh, Aceh, Indonesia

1. Abstract (minor revision)

 In the conclusion section, significant results have been attached to preventive behavior and community perceptions of the severity of COVID-19 disease, but to support objective data, it is necessary to attach data in the form of accurate numbers in accordance with the findings in the field. In the results section, you mentioned that there is a relationship between self efficacy and perceived influence on preventive behavior. How did you come to the conclusion that these variables are related?

 2. Methods (major revision)

 This is an interesting study because it is a large-scale study of the country of Indonesia. I have high hopes that this research will be successfully indexed so that it can be used as a reference by other studies in information about *Community preventive behavior and perception on the severity of COVID-19 disease in Indonesia*. As with other research articles that have been published in various journals, of course your research article needs to be improved to make it worth reading.

 Your research obtained data from a google form that was distributed through various social media. You managed to get thousands of responses. But I am curious how can you ensure that one respondent does not fill in the google form more than once? Is there strict control over the filling of google forms by respondents? I hope that your data does not become biased because of this.

 Then you mentioned that the respondents were spread across Indonesia. How do you ensure that the respondents are indeed spread evenly across Indonesia, which consists of 38 provinces? Perhaps you have your own reference in determining the distribution throughout Indonesia as you mentioned, please explain.

 3. Study and Sample (minor revision)

 This research expands to the Indonesian public with contributions from social media influencers. How do you ensure this research is representative of all Indonesians? Respondents participating in your research are Indonesian citizens. How do you ensure that the people filling in the data are Indonesian citizens? What if a foreigner residing in Indonesia receives and fills out a questionnaire from your research, how do you know?

 4. Conceptual Model (major revision)

 This research uses HBM as the main reference. What should be a common concern in using SEM must be based on a certain theory. This is because the function of SEM is to confirm the model formed on real data obtained in the field. In addition, the determination of the direction of the relationship built in the model must be theoretically corroborated. This will also affect the determination of the amount of data or research samples that are suitable for use in SEM and feasible in terms of sample adequacy in testing a theory-based model.

 5.  Minor revision

 ​Correct the English writing errors in the article. I have marked them on the linked annotated article. For example, in the questionnaire table in the *methods section*, and in the *discussion *section. The COVID-19 incidence rate has been mentioned from mid-2021 to the end of 2021. To add to the accuracy of this study, the incidence rate of COVID-19 until 2022 should also be attached.

Is the work clearly and accurately presented and does it cite the current literature?

Yes

If applicable, is the statistical analysis and its interpretation appropriate?

Partly

Are all the source data underlying the results available to ensure full reproducibility?

Yes

Is the study design appropriate and is the work technically sound?

Yes

Are the conclusions drawn adequately supported by the results?

Partly

Are sufficient details of methods and analysis provided to allow replication by others?

Yes

Reviewer Expertise:

Nutrition Department

I confirm that I have read this submission and believe that I have an appropriate level of expertise to confirm that it is of an acceptable scientific standard, however I have significant reservations, as outlined above.
